# Reducing Treatment Burden Among People With Chronic Conditions Using Machine Learning: Viewpoint

**DOI:** 10.2196/29499

**Published:** 2022-02-10

**Authors:** Harpreet Nagra, Aradhana Goel, Dan Goldner

**Affiliations:** 1 One Drop New York, NY United States; 2 Integrated Care Bayer Pharmaceuticals San Francisco, CA United States

**Keywords:** artificial intelligence, machine learning, behavioral science, chronic conditions, self-care, behavior, chronic disease, prediction, algorithm, lifestyle, adapt

## Abstract

The COVID-19 pandemic has illuminated multiple challenges within the health care system and is unique to those living with chronic conditions. Recent advances in digital health technologies (eHealth) present opportunities to improve quality of care, self-management, and decision-making support to reduce treatment burden and the risk of chronic condition management burnout. There are limited available eHealth models that can adequately describe how this can be carried out. In this paper, we define treatment burden and the related risk of affective burnout; assess how an eHealth enhanced Chronic Care Model can help prioritize digital health solutions; and describe an emerging machine learning model as one example aimed to alleviate treatment burden and burnout risk. We propose that eHealth-driven machine learning models can be a disruptive change to optimally support persons living with chronic conditions.

## Introduction

The COVID-19 pandemic has surfaced multiple concerns present within our health care systems, including the high infection risk prevalent among people with chronic conditions, and the fact that practitioners can only provide specialized responses to acute illnesses [1]. These, in turn, leave people with chronic conditions to experience fragmented, poorly coordinated, and limited support in their treatment [2], which exacerbates the treatment burden patients experience as they encounter decreased support for their ongoing medical care [3]. Increased treatment burden can heighten the risk for illness-related burnout—a chronic affective state comprising symptoms of emotional exhaustion, physical fatigue, and cognitive weariness, often the outcome of depletion of energetic resources resulting from prolonged exposure to medical distress [4]. Considered the most efficacious of the various chronic illness frameworks [5], the Chronic Care Model (CCM) [6,7] addresses how health care teams, including physicians, can better support those with chronic conditions by shifting care focus to coordinated self-management and decision-making support [8-10].

eHealth supports individuals in self-care and facilitates interactions and collaboration within the health care system, thereby reinforcing the value of the CCM. eHealth technologies are connective elements that build bridges between stakeholders in the ecosystem [11]. The eHealth enhanced Chronic Care Model (eCCM), developed in 2015 by Gee et al [12], is a framework that incorporates eHealth literature into the CCM components and promotes understanding how eHealth tools such as mobile health (mHealth) apps, machine learning (ML), e-communities, electronic health records, and eHealth education may facilitate the implementation of the CCM in a digital space (eg, by enabling self-tracking of health data, empowering involvement in shared decision making, supporting preparation for appointments, and enabling personalized decision support through visualization of data and reminders). Thus, there are various technologies that have been suggested to improve the functionality of the different components of the CCM.

Of the suggested technologies, ML offers new opportunities to deliver more accessible, equitable, personalized, and cost-efficient chronic care programs. ML may help mitigate treatment burden and burnout risk by providing self-management and decision-making interventions that guide and support people with chronic conditions. This guidance and support can be delivered directly or via a mobile app; it can also be provided to a coach or care provider to consider when working with a person with chronic conditions.

Here, we introduce an emerging ML model called “the outcomes model” as an example of the eCCM framework, which integrates behavior and data science principles to reduce treatment burden and chronic-condition–related burnout. The outcomes model correlates an individual's health outcomes months in advance with current lifestyle and biometric markers and could be used to help determine which kinds of lifestyle activities (eg, maintaining a sleep routine or adding more daily physical activity) are most likely to benefit an individual at a given time. It may not only help people make meaningful lifestyle decisions but may also enhance their motivation through self-monitoring and feedback on health behaviors and outcomes in order to complete activities necessary to improve chronic-condition–related self-management.

## Machine Learning at One Drop

One Drop is a mobile app that passively and actively collects data (passively from wearables such as Apple Watch and FitBit, and actively from user-inputted information directly into the mobile app). It provides ML health trend prediction, tailored educational content, and personalized health coaching services for people who want to prevent and manage chronic conditions, such as diabetes and heart disease. Upon downloading the app, members actively grant permission to use the data only for app improvements and for research and development of ML models for use within the app.

One Drop data collection spans 195 countries, and as of this writing, the data comprise over 30 billion data points. Some of the categories and their components are presented in the following:

1. Biometrics data: heart rate, blood pressure, skin temperature, and blood glucose concentration.
2. Behavior data: physical activity, sleep, food and mealtimes, medications and consistency of medication use, and geographic movement.
3. Outcome data: laboratory measures, such as glycosylated hemoglobin type A1c (HbA1c) and cholesterol levels, and self-reported metrics, such as weight, body mass index, and waist circumference.
4. Engagement data: app use, interactions with coaches, use of educational resources, interactions with peer support networks, response rate to app notifications, and others.

Information is collected on multiple cadences; some wearable data are collected passively, while others are set by the person logging manually. App features call for medical-condition–specific subsampling strategies. More frequent data input supports more accurate outcomes forecasts.

Currently, ML algorithms trained on these data predict health outcomes, such as weight, average blood glucose concentration (HbA1c), time-in-range, or blood pressure, using the inputs described above (eg, biometrics, behavior, outcomes, and engagement), for 1-6 months in advance. One Drop data from a sample of over 50,000 app users were used to train a suite of patent pending supervised learning models, each for a different health metric (weight, blood pressure, or blood glucose) and time horizon (1-2 months, 2-3 months, 3-4 months, or 4-6 months). Data collected prior to 2019 were used to train the algorithms; data from January 2019 through February 2020 were used for testing. Test set predictions were 10%-40% more accurate than a naïve (benchmark) prediction of “no change.” Further details of that work have been presented previously [13,14], and model development is still in progress. For the present discussion, we note this as an example that informative biometric predictions based in part on behavioral inputs exist today. However, predictions such as these cannot in and of themselves produce improved outcomes nor reduce treatment burden. Only in combination with a behavioral support framework can such benefits be realized. We next describe such a combination, which we call the outcomes model.

## The Outcomes Model

For people with chronic conditions, medical appointments with health care providers may occur on a quarterly or biannual basis. Having a system such as the outcomes model could provide lifestyle support in between medical visits, while simultaneously giving people with chronic conditions a predictive insight into how their habits are impacting their overall health. To date, no eHealth offerings focused on people with chronic conditions integrate a combination of multiple ML models to (1) forecast outcomes up to 6 months into the future; (2) provide insight into which lifestyle behavior modification may most significantly impact a person’s desired clinical outcome; and (3) use various data inputs (eg, biometrics, behavior, outcomes, and engagement) to support behavior change and reduce treatment burden for people with chronic conditions.

The hypothesis we are exploring is how to use an ML model capable of predicting likely changes in outcomes to reduce treatment burden within the eCCM. A potential approach is to use a forecasting model to determine which lifestyle modifications are most likely to yield the greatest improvement on forecasted clinical outcomes of interest (eg, weight, HbA1c, blood pressure, and time-in-range). Based on the forecast of various health outcomes-focused interventions, an optimal current lifestyle modification focus could be selected. A combination of human and automated interventions could then be initiated to suggest adjustments to the individual’s behavior. After an initial trial period to evaluate the effect of a suggestion, the guidance could be recalculated with up-to-date information, and the focus might either be maintained or switched to a new, now most optimal, choice. If the recommended focus is not practical to be addressed by people with chronic conditions for any reason, the next most effective mode could be selected. In this way, the system can support people with chronic conditions in making progress toward better health outcomes using tailored interventions that adapt to each individual, evolve with time, and are informed by their predicted effect on the individual’s health.

Predictive guidance, developed as described above, might be digitally delivered directly to people with chronic conditions, which can support the calculated guidance provided through a healthcare professional. This predictive guidance could reduce treatment burden by helping people with chronic conditions seek additional help from a health care professional. The information from predictive guidance could be used more broadly as a part of a health care network’s decision support system. Prescribers would be required to evaluate potential medical interventions such as medication dose changes. Lifestyle modifications, such as modifying physical activity or carbohydrate intake, could be improved upon by a digital health coach, a dietitian, or other subject matter experts. These coaches are typically more readily available for appointments between clinic visits, and the use of their services may reduce the burden on the health care system by increasing patient engagement in self-care and supporting health care providers with additional patient health insight.

## Discussion

### Machine Learning-Based Lifestyle Modifications

The general idea of informing chronic condition management with computed forecasts about an individual is receiving increasing attention. Schwartz et al [15] review a variety of current developments based on the rise in quantification of many aspects of daily health. One of the many features they see as important in the evolution of digital health is prioritization of interventions, and in that light, they review concepts such as ecological momentary interventions that “assess the person’s (digital twin) status and the model delivers interventions as needed, when needed—perhaps even preemptively.” More recently, Chevance et al [16] have elaborated on emerging applications that use predictions to support behavior change, with examples including predictions of smoking or walking behaviors. One aspect of their discussion centers on continuous tuning interventions, that “include real-time optimization algorithms, which [based on such forecasts] can further adjust intervention content or delivery aspects to the needs of a specific individual.” Increased attention notwithstanding, we concur with the assessment by Chevance et al that “Computational models and associated computerized simulations are still relatively under-used in the behavior change field.”

A wide set of ideas is discussed in those papers, and we are broadly supportive of all of them. The approach we have described here sits squarely among those ideas. While Schwartz et al [15] describe the role of algorithms for informing a digital phenotype, their digital phenotype is not necessarily predictive of a future state. Chevance et al [16] flesh out continuous tuning based on updated predictions, but their examples focus on behavioral interventions tuned via behavioral predictions. Our aim here is to advocate specifically for placing biometric predictions in a central role in steering behavioral interventions. This stance is tantamount to a hypothesis that a dominant contributor to treatment burden for people with chronic conditions is uncertainty. Will a particular behavior change succeed in improving my condition, given my history and circumstances? Is the work I am doing now truly going to make a difference later? We believe that definitive answers to those personal questions, updated as individual behaviors, the chronic condition and circumstances evolve, can make a substantial reduction in treatment burden. We further believe that predicting the biometric effect of candidate behavioral interventions to each specific individual is the most promising way to obtain those answers.

Here, we have described a strategy in which a biometric forecasting model is used to select the lifestyle intervention most likely (at a given moment) to improve an individual’s forecast, and to use that as the basis for informing interventions. We acknowledge that this strategy is not a straightforward exercise. People with chronic conditions do not typically have the privilege to make one behavior change at a time. They must instead focus on making multiple changes (eg, diet, physical activity, and medication) simultaneously. Type 1 diabetes provides a good case in point for this issue. If a person living with type 1 diabetes decides to engage in more physical activity, they must make simultaneous changes to carbohydrate intake, as well as adjusting insulin-to-carbohydrate ratios. In this scenario, a suggested ML model approach is to observe the population of mobile app users for typical correlations with changes to behavior; the next step is to apply correlated factors to hypothetical changes of the past 30 days of an individual’s observed behavior and examine if there are any resulting changes to the forecasted outcome. It is, to be sure, more difficult to learn from data where many recorded behaviors are correlated and overlapping, calling for careful statistical evaluation of whether the resulting forecast changes differ significantly. In these circumstances, the results and recommended behaviors can be less specific and more complex—less than ideal if the goal is to reduce treatment burden and burnout by making clearer recommendations that are more likely to succeed. Nonetheless, whether choosing a single behavior change or a combination, knowing which choice is forecasted to result in greater improvement for a given individual could inform both guidance and goal setting for that individual. Such a result would not constitute proof or a guaranteed outcome. However, the vast data reservoir on which the ML model is trained offers the possibility of learning which interventions have led to greater improvements among people in similar circumstances to the individual in question.

While recognizing the challenges, we believe that relieving treatment burden cannot improve beyond a limited threshold without reducing uncertainty about the effectiveness of a behavioral intervention for each individual at each moment in time. Fusing biometric prediction with behavioral science frameworks such as eCCM is, in our view, the best strategy for reducing that uncertainty. Whether through a predictive guidance system such as the outcomes model described above or in some other form, adjustments to behavioral interventions must be informed by an individualized prediction of which adjustments are most likely to succeed. Adjustments so informed are necessary for sustainable chronic illness prevention, management, and hopefully treatment burden alleviation.

### Conclusion

ML-based biometric predictions used in the context of established behavior change frameworks offer exciting potential to support and reduce treatment burden, as well as mitigate burnout risk for those living with chronic conditions. Chronic care management requires constant attention, which necessitates deep engagement with supportive tools. eHealth solutions such as the outcomes model may break down the boundaries that define traditional, nondigital care. Such innovation should support digital health care’s progression out of reactive and into proactive chronic care.

## Abbreviations

CCM: Chronic Care Model
eCCM: eHealth enhanced Chronic Care Model
HbA1c: glycosylated hemoglobin, type A1C
mHealth: mobile health
ML: machine learning
